# Zen meditation, Length of Telomeres, and the Role of Experiential Avoidance and Compassion

**DOI:** 10.1007/s12671-016-0500-5

**Published:** 2016-02-22

**Authors:** Marta Alda, Marta Puebla-Guedea, Baltasar Rodero, Marcelo Demarzo, Jesus Montero-Marin, Miquel Roca, Javier Garcia-Campayo

**Affiliations:** Miguel Servet University Hospital, University of Zaragoza, Zaragoza, Spain; Instituto Aragonés de Ciencias de la Salud. Red de Investigación en Atención Primaria (REDIAPP), Zaragoza, Spain; Centro Rodero: Clinica de Neurociencias, Santander, Spain; University Federal of Sao Paulo (UNIFESP), Sao Paulo, Brazil; Institut Universitari d’Investigació en Ciències de la Salut, Palma de Mallorca, Illes Balears Red de Actividades Preventivas y Promoción de la Salud en Atención Primaria (RediAPP), Universitat de les Illes Balears, Illes Balears, Spain; Miguel Servet University Hospital, Avda Isabel La Catolica 1, 50009 Zaragoza, Spain

**Keywords:** Telomere length, Mindfulness, Experiential avoidance, Compassion

## Abstract

Mindfulness refers to an awareness that emerges by intentionally focusing on the present experience in a nonjudgmental or evaluative manner. Evidence regarding its efficacy has been increasing exponentially, and recent research suggests that the practice of meditation is associated with longer leukocyte telomere length. However, the psychological mechanisms underlying this potential relationship are unknown. We examined the telomere lengths of a group of 20 Zen meditation experts and another 20 healthy matched comparison participants who had not previously meditated. We also measured multiple psychological variables related to meditation practice. Genomic DNA was extracted for telomere measurement using a Life Length proprietary program. High-throughput quantitative fluorescence in situ hybridization (HT-Q-FISH) was used to measure the telomere length distribution and the median telomere length (MTL). The meditators group had a longer MTL (*p* = 0.005) and a lower percentage of short telomeres in individual cells (*p* = 0.007) than those in the comparison group. To determine which of the psychological variables contributed more to telomere maintenance, two regression analyses were conducted. In the first model, which applied to the MTL, the following three factors were significant: age, absence of experiential avoidance, and Common Humanity subscale of the Self Compassion Scale. Similarly, in the model that examined the percentage of short telomeres, the same factors were significant: age, absence of experiential avoidance, and Common Humanity subscale of the Self Compassion Scale. Although limited by a small sample size, these results suggest that the absence of experiential avoidance of negative emotions and thoughts is integral to the connection between meditation and telomeres.

## Introduction

Telomeres are DNA and protein complexes that are located at the end of linear chromosomes and are necessary for the complete replication of DNA as well as chromosome stability. Intact telomeres protect chromosomes from nuclease degradation, end-to-end fusion, and cellular senescence (Blackburn 2000). In general, telomeres shorten with age and can serve as an early predictor of the onset of several diseases, including hypertension (Demissie et al. 2006), atherosclerosis (Samani, Boultby, Butler, Thompson, and Goodall 2001), type 2 diabetes mellitus (Sampson, Winterbone, Hughes, Dozio, and Hughes 2006; Zee, Castonguay, Barton, Germer, and Martin 2010), cancer mortality (Willeit et al. 2010), cardiovascular disease (Epel et al. 2009a, b), and cognitive decline and dementia (Martin‐Ruiz et al. 2006).

Interestingly, from a psychological perspective, telomere shortening can be accelerated by several behavioral factors, including poor diet (Nettleton, Diez-Roux, Jenny, Fitzpatrick, and Jacobs 2008; Valdes et al. 2005), poor sleep (Prather et al. 2011), cigarette smoking (Valdes et al. 2005), excessive alcohol consumption (Pavanello et al. 2011), sedentary lifestyle (Cherkas et al. 2008), and several psychological factors, such as personality characteristics (O’Donovan et al. 2009), psychiatric disorders (Lindqvist et al. 2015), and psychological distress (Shalev et al. 2013). For example, a reference study (Epel et al. 2004) compared the lengths of the telomeres in the white blood cells of mothers of chronically ill children with the telomere lengths of mothers of healthy children. The longer a woman had been the primary caregiver for her ill child (the children’s conditions ranged from gut disorders to autism), the shorter were her telomeres. Moreover, in both groups, the more severe a woman’s psychological stress was, the shorter her telomeres were. The reduction in telomere lengths of the most stressed mothers (compared with the reduction in the least stressed mothers) was equivalent to that caused by at least a decade of ageing.

It has been suggested that healthy lifestyle factors can promote telomerase (the cellular enzyme that adds telomeric repeat sequences to the ends of chromosomal DNA, preserving not only telomere length but also healthy cell function and long-term immune function), telomere maintenance or even lengthening. These factors include physical exercise (Puterman et al. 2010), a body mass index <25 kg/m^2^ (Sun et al. 2012), not smoking (O’Donnell et al. 2008), and healthy diet (Paul 2011).

Meditation has also been proposed to be a healthy lifestyle factor that affects telomere length. Recent empirical studies have shown a positive association between meditation and longer telomeres (Hoge et al. 2013a, b) as well as an increase in telomerase (Schutte and Malouff 2014), suggesting that meditation may play an important role in preventing illnesses. However, one of the most challenging questions to answer is how the practice of meditation is related to telomere dynamics.

The aim of this study was first to replicate and strengthen the hypothesis that meditation is associated with longer telomeres. Only a few studies have demonstrated this relationship (Schutte and Malouff 2014; Epel et al. 2009a, b; Conklin et al. 2015; Jacobs et al. 2011). Second, to elucidate the psychological mechanisms underlying this potential relationship, we compared a group of expert meditators with a matched comparison group and used several questionnaires to assess different psychological constructs related to meditation that could be involved in delaying the ageing process. To measure telomere length, we employed the HT Q FISH technique, a unique method that not only determines the MTL but also the percentage of short telomeres in individual cells, which indicates the extent of cellular ageing.

## Method

### Participants

The group of expert meditators (*N* = 20) was recruited from the Soto Zen Spanish Buddhist community. The healthy matched comparison group (*N* = 20) was recruited from healthy relatives and friends of the meditators who had a similar lifestyle and were matched by gender, age (±2 years) and ethnic group. Participants in both groups had to be aged between 18 and 65 years of age and be fluent in Spanish (to answer the study questionnaires) to be included in the study. Individuals were excluded from the study if they had history or current diagnosis of a psychiatric disorder based on the MINI interview, were receiving pharmacological or psychological treatment, or were suffering from severe medical disorders that could affect telomerase activity (such as any type of cancer or AIDS). Additionally, the meditators were required to have practiced continuous meditation for more than 10 years before the start of the study (including the last 10 years) with a mean of at least 60 min/day of formal practice during the entire period. Soto Zen meditation is a sitting meditation that focuses on the act of breathing. Zen meditation is a form of open monitoring meditation. The study was approved by the Aragon Ethics Committee and was performed in accordance with the ethical standards of the 1964 Declaration of Helsinki. All of the participants provided their written informed consent before participating in the study.

### Procedures

The two groups were selected and provided their signed consent upon arriving at the first study visit. In the morning, all of the subjects submitted their blood samples for telomere measurements. They then completed a battery of sociodemographic, psychological, and health-related questionnaires.

### Measurements

#### Telomere Measurement

In brief, cells were plated in 384-well plates, fixed with methanol/acetic acid (3/1, *v*/*v*), treated with pepsin to digest the cytoplasm and the nuclei were then processed for hybridisation in situ with a fluorescent Peptide Nucleic Acid probe (PNA) that recognizes telomere repeats (sequence: Alexa488-OO-CCCTAACCCTAACCCTAA, Panagene). After several washing steps, as described by standard DAPI incubation procedures for DNA staining, the wells were filled with mounting medium, and the plate was stored overnight at 4 °C.

Quantitative image acquisition and analysis was performed on a High Content Screening Opera System (Perkin Elmer) using the Acapella software, version 1.8 (Perkin Elmer, Waltham, MA, USA). Images were captured using a ×40 0.95 NA water immersion objective. UV and 488-nm excitation wavelengths were used to detect the DAPI and A488 signals, respectively. The results of the intensity of each foci identified were exported from the Acapella software (Perkin Elmer). The telomere length distribution and median telomere length were calculated with a proprietary program from Life Length.

Blood was obtained by venipuncture from the antecubital vein and collected into Sodium Heparin tubes. The blood samples were processed within 24 h of extraction to isolate peripheral blood mononuclear cells (PBMCs) by a Ficoll-Hypaque gradient. The PBMCs were rinsed in phosphate buffer solution, counted and resuspended at 10 million cells per milliliter in a freezing medium. Aliquots were frozen at −80 °C and placed in liquid nitrogen for storage.

#### Health-Related and Psychological Variables

Labco has developed a comprehensive health questionnaire that is mandatory for all of the participants included in telomere studies. It is available from Labco upon request. The questionnaire includes questions on the following variables: cardiovascular disorders, neurodegenerative disorders, infectious disorders, autoimmune disorders, and other disorders. Other topics assessed in the questionnaire include the use of different medications and characteristics of the participant’s diet.

#### Sociodemographic Characteristic and Meditation Data

Information on participant’s gender, age, ethnic group, marital and socioeconomic status, and years of education were collected. Experience with meditation was also assessed. The mean weekly meditation time was homogeneous in the Zen group with a median of 90 min per day. Therefore, we assessed the number of months that the participants had practiced meditation over their lifetime, noting the periods in which their practice had been discontinued.

#### The Mini-International Neuropsychiatric Interview (MINI)

(Sheehan et al. 1998). The MINI is a short, structured diagnostic interview that is used for the diagnosis of DSM-IV and ICD-10 psychiatric disorders. With an administration time of approximately 15 min, the MINI was designed to provide a short but accurate structured psychiatric interview for multicenter clinical trials and epidemiology studies. The MINI has a validated Spanish version (Ferrando et al. 1998).

#### The Mindful Attention Awareness Scale (MAAS)

(Brown and Ryan 2003). The MAAS is a 15-item scale designed to assess a core characteristic of dispositional mindfulness, namely open or receptive awareness of and attention to what is occurring in the present. The scale has strong psychometric properties and has been validated with college, community, and cancer patient samples. There is a Spanish version of the MAAS that has displayed adequate psychometric properties (Soler Ribaudi et al. 2012) (Cronbach’s α = 0.89).

#### The Five Facet Mindfulness Questionnaire (FFMQ)

(Baer, Smith, Hopkins, Krietemeyer, and Toney 2006). This questionnaire consists of 39 items that assess five facets of mindfulness. The items are rated using a Likert scale ranging from 1 (never or very rarely true) to 5 (very often or always true), with higher scores meaning higher self-reported mindfulness skills. The five facets assessed are observing (noticing or attending to internal and external experiences, such as sensations, thoughts, or emotions); describing (labeling internal experiences with words); acting with awareness (focusing on one’s activities at a given moment as opposed to behaving mechanically); nonjudging of inner experience (taking a nonevaluative stance towards thoughts and feelings); and nonreactivity to inner experience (allowing thoughts and feelings to come and go without getting caught up in them or being carried away by them). The Spanish version of the FFMQ has been validated and shows good psychometric properties (Cebolla et al. 2012). The Cronbach’s α of the five subscales was 0.81 for observing, 0.91 for describing, 0.89 for acting with awareness, 0.91 for nonjudging, and 0.80 for nonreactivity.

#### Experiential Avoidance (AAQ-II)

(Bond et al. 2011). The second version of the Acceptance and Action Questionnaire was used to measure experiential avoidance. The AAQ-II contains seven items that assess experiential avoidance. The items examine one’s unwillingness to experience emotions and thoughts (e.g., “I am afraid of my feelings”) and the inability to be in the present moment and engage in valued behavior when unwanted emotions or thoughts are present (e.g., “My painful memories prevent me from having a fulfilling life”). The participants indicated their response to each question using a seven-point scale (1 = never true to 7 = always true). The AAQ-II is scored by summing all of the items, and higher scores correspond to greater experiential avoidance. Previous research has demonstrated that the AAQ-II has good psychometric properties. This instrument also has a translated and validated Spanish version (Ruiz et al. 2013) (Cronbach’s α = 0.75–0.93).

#### The Self-Compassion Scale (SCS)

(Neff 2003). The SCS has 26 items measuring six components of self-compassion (negative aspects are reverse coded): Self-kindness (e.g., “When I’m going through a very hard time, I give myself the caring and tenderness I need”), self-judgment (e.g., “I’m disapproving and judgmental about my own flaws and inadequacies”), common humanity (e.g., “I try to see my failings as part of the human condition”), isolation (e.g., “When I fail at something that’s important to me, I tend to feel alone in my failure”), mindfulness (e.g., “When something upsets me, I try to keep my emotions in balance”), and over-identification (e.g., “When I’m feeling down, I tend to obsess and fixate on everything that’s wrong”) (Ruiz et al. 2013). Adequate psychometric properties have been reported for this scale (Ruiz et al. 2013). The items are rated on a five-point response scale ranging from 1 (almost never) to 5 (almost always). A Spanish version of the scale has also been developed (Garcia-Campayo et al. 2014). The Cronbach’s α of the 26-item SCS was 0.87 (95 % CI = 0.85–0.90) and ranged between 0.72 and 0.79 for the six subscales.

Resilience was evaluated using the ten-item Connor-Davidson Resilience Scale (CD-RISC) (Campbell-Sills and Stein 2007), a self-administered questionnaire that is designed as a Likert type additive scale with five response options (0 = never to 4 = almost always), which has a single dimension in the original version. The final score on the questionnaire is the sum of the responses obtained from each item (range, 0–40), and the highest score indicates the highest level of resilience. The Spanish version of the scale has been recently validated and showed appropriate psychometric parameters (Notario-Pacheco et al. 2011) (Cronbach’s α = 0.85).

#### The Hospital Anxiety and Depression Scale (HADS)

(Zigmond and Snaith 1983). The HADS is a 14-item questionnaire with seven items measuring anxiety (HAD-A) and seven measuring depression (HAD-D). The ratings are totaled to obtain a score ranging from 0 to 21 for anxiety and depression, with higher scores indicating greater depression or anxiety. Scores between 8 and 10 represent possible cases of anxiety or depression, and scores that are ≥11 correspond to probable cases. The validity and reliability of the Spanish version of the HADS have been shown to be good (Herrero et al. 2003) (The Cronbach’s α of the anxiety and depression subscales was, respectively, α = 0.84 and α = 0.85).

Life satisfaction was measured using the Satisfaction with Life Scale (SWLS) (Diener et al. 1985). The scale consists of five statements (e.g., “In most ways, my life is close to my ideal”), and the participants rate whether they agree or disagree with each statement using a five-point Likert scale. Overall life satisfaction (using a scale from 5 to 25) is calculated as the sum of the responses to all of the items; a higher score indicates a higher level of life satisfaction. Previous studies have consistently demonstrated a high reliability and a single-factor structure of SWLS items. This instrument has a translated and validated Spanish version that has shown adequate parameters (Vázquez et al. 2013) (Cronbach’s α = 0.86).

Subjective happiness was measured using the SHS (Lyubomirsky and Lepper 1999), which is a four-item measure of subjective global happiness rated on a seven-point Likert scale. The current study used the Spanish version of the SHS, which has established validity (Extremera and Fernández-Berrocal 2014). A single SHS score is the mean of the responses to the four items. The SHS scores can range from 1 to 7, where a higher score indicates a higher level of happiness (Cronbach’s α = 0.68–72).

### Data Analyses

First, depending on their nature, all of the variables were described using either mean and standard deviation (SD) values or percentages. The comparisons between groups were performed using Student’s *t* test and a chi-squared test. The effect sizes on telomere length between meditators and nonmeditators were assessed using Cohen’s *d*. The descriptive statistics and raw Pearson correlations (*r*) between the sociodemographics, the psychological variables, and the two telomere measurements were calculated considering the total sample. The sociodemographic and psychological variables that showed significant results in the raw analysis were included in two multivariate linear regression models that assessed the adjusted relationships between the telomere measurements and psychological outcomes. We used the stepwise method to introduce the variables into the regression models and to assess the adjusted relationships between the telomere measurements and psychological outcomes because of the small sample size (*n* = 40) and the subsequent problems with statistical power. Standardized coefficients (beta) were used to assess the individual contribution of the variables in explaining the telomere length, and the Wald test was used to evaluate the significance of the variables. The adjusted multiple determination coefficients (adj-*R*^2^) were also calculated to observe their grouped explanatory power, and their significance was assessed using analysis of variance (Martínez-González et al. 2006). The partial correlation coefficients (*R*) were calculated, which indicated the correlation between two variables when the effects of the other variables in the equation were removed. The Kolmogorov-Smirnov (KS) test was used to determine whether the conditional distribution of the residuals met the assumption of normality. Finally, it was confirmed that the Durbin-Watson values (DW) approached a value of approximately 2.00 to rule out autocorrelation problems in the errors. All of the tests used were bilateral, and the significance level was α < 0.05. SPSS-19 statistical software package was used.

## Results

One meditator was excluded from the analyses because he had prostate cancer, and two individuals in the comparison group were excluded because they were being treated with antidepressants. The sociodemographic and health characteristics of the sample are summarized in Table 1. There was a predominance of middle-aged elderly European men. Gender, age, and ethnic group variables were matched to avoid significant differences. There were no differences between the samples in other sociodemographic variables (living with a partner and years of education), most health habits (tobacco, alcohol and medication consumption and vegetarian diet), and several chronic medical disorders. The only differences between the groups were in the average body mass index (BMI), which was significantly higher in the meditators and showed mild overweight (BMI >25), and the amount of physical exercise, which was lower in the meditators. The mean period of daily meditation reported by the meditators was 75 min with a standard deviation of 15 min. The mean length of time that the participants had practiced meditation over their lifetime was 180 months with a standard deviation of 12 months. Both of the measures were rounded by the participants because it was difficult to provide more accurate data, resulting in quite homogenous data.

**Table 1:** Sociodemographic and health characteristics of the sample

Variables	Meditators	Nonmeditators	Significance
Sociodemographics			
Gender (male)^a^	14/20 (70 %)	14/20 (70 %)	*X* ^2^ = 0.0; *df* = 1; *p* = 1
Age (mean, SD)^a^	48.55 (8.05)	48.30 (8.76)	*t* = 0.094; *df* = 38; *p* = 0.926
Ethnic group (white)^a^	20/20 (100 %)	20/20 (100 %)	*X* ^2^ = 0; *df* = 1; *p* = 1
Live with partner (%)	17/20 (85 %)	18/20 (90 %)	*X* ^2^ = 0.229; *df* = 1; *p* = 0.663
Years of education (mean, SD)	14.25 (4.37)	12.60 (3.11)	*t* = 1.37; *df* = 38; *p* = 0.178
Healthy habits			
Tobacco (>10 cigarettes/day)	3/20 (15 %)	5/20 (25 %)	*X* ^2^ = 0.625; *df* = 1; *p* = 0.429
Alcohol^b^	0/20 (0 %)	0/20 (0 %)	*X* ^2^ = 0; *df* = 1; *p* = 1
Medications	0/20 (0 %)	0/20 (0 %)	*X* ^2^ = 0; *df* = 1; *p* = 1
Vegetarian diet	0/20 (0 %)	0/20 (0 %)	*X* ^2^ = 0; *df* = 1; *p* = 1
Body mass index (mean, SD)	25.25 (1.50)	23.79 (1.96)	*t* = 2.63; *df* = 38; *p* = 0.012*
Exercise (>3 h/week)	1/20 (5 %)	7/20 (35 %)	*X* ^2^ = 5.65; *df* = 1: *p* = 0.048*
Medical disorders			
Diabetes	1/20 (5 %)	3/20 (15 %)	*X* ^2^ = 1.111; *df* = 1; *p* = 0.292
Hypertriglyceridemia	1/20 (5 %)	3/20 (15 %)	*X* ^2^ = 1.111; *df* = 1; *p* = 0.292
Hypercholesterolemia	1/20 (5 %)	3/20 (15 %)	*X* ^2^ = 1.111; *df* = 1; *p* = 0.292
Hypertension	3/20 (15 %)	4/20 (20 %)	*X* ^2^ = 0.173; *df* = 1; *p* = 0.677
Arthrosis	1/20 (5 %)	3/20 (15 %)	*X* ^2^ = 1.111; *df* = 1; *p* = 0.292

*p < 0.05

^a^Matched variables

^b^>4 units/day for men or >3 units/day for women

The other health variables included in the Labco health questionnaire were not summarized in the table due to space constraints; however, they did not show significant differences between the groups. These variables included the prevalence of cardiovascular disorders (heart infarction, atherosclerosis, arrhythmia, and varicose veins), neurodegenerative disorders (Alzheimer’s disease, Parkinson’s disease, multiple sclerosis, and lateral amyotrophic sclerosis), infectious disorders (syphilis, hepatitis B and C, mononucleosis, toxoplasmosis, and cytomegalovirus), autoimmune disorders (psoriasis, rheumatoid arthritis, and lupus erythaematosus), and other disorders (osteoporosis, chronic obstructive pulmonary disease, lung fibrosis, congenital dyskeratosis, progeria, aplastic anaemia, asthma, and allergies). Other variables assessed were the use of different medications (such as oestrogens, thyroid hormone agents, and antioxidants) and characteristics of the participant’s diet (such as consumption of fruits, vegetables, red meat, and fat).

### Telomere Measurement

A *t* test showed that the expert meditators group (mean = 10.82 kb; SEM = 0.23; SD = 1.03) had a significantly longer MTL (*t* = 2.97; *df* = 38; *p* = 0.005; Cohen’s *d* = 0.94) compared with the comparison group (mean = 9.94 kb; SEM = 0.19; SD = 0.84).

The data concerning the 20th percentile were similar, showing that the prevalence of short telomeres in the cells of the expert meditators group (mean = 5.22 kb; SEM = 0.11; SD = 0.48) was significantly lower (*t* = 2.84; *df* = 38; *p* = 0.007; Cohen’s *d* = 0.91) than the comparison group (mean = 4.80 kb; SEM = 0.10; SD = 0.44).

### Mindfulness Variables

As expected, the expert meditators showed significantly better results in the measurements that were related to mindfulness abilities, such as attention and awareness, observing, describing, nonjudging, resilience, self-compassion, and satisfaction with life and subjective happiness. Moreover, the expert meditators reported significantly lower experiential avoidance, anxiety, and depression (Table 2).

**Table 2:** Psychological variables in meditators (*n* = 20) and nonmeditators (*n* = 20)

Variable	Meditators, mean (SD)	MTL (*r*)	20th (*r*)	Nonmeditators, mean (SD)	MTL (*r*)	20th (*r*)	t (*df*)	MTL (*r*)^a^	20th (*r*)^a^
MAAS	4.50 (0.16)	0.09	0.15	3.39 (0.26)	0.44	0.43	15.79 (38)***	0.49**	0.49**
FFMQ Observing	28.65 (1.42)	0.29	0.11	23.95 (0.99)	−0.10	−0.05	12.08 (38)***	0.45**	0.39*
FFMQ Describing	26.90 (1.33)	−0.46*	−0.42	29.25 (1.29)	−0.13	−0.05	−5.66 (38)***	−0.50**	−0.45**
FFMQ Acting aware	32.55 (1.50)	−0.35	−0.28	27.75 (1.44)	−0.01	0.12	10.28 (38)***	0.46**	0.40*
FFMQ Nonjudging	31.65 (1.46)	0.20	0.23	26.85 (4.46)	−0.15	−0.06	4.57 (38)***	0.23	0.26
FFMQ Nonreacting	22.95 (2.50)	−0.35	−0.29	21.35 (2.41)	−0.07	−0.06	2.05 (38)*	−0.05	−0.02
AAQ2	14.75 (2.82)	−0.41	−0.41	22.60 (1.69)	−0.23	−0.09	−10.64 (38)***	−0.53***	−0.49**
HADS Anx	0.75 (0.63)	0.30	0.30	4.05 (0.75)	−0.48*	−0.47*	−14.87 (38)***	−0.43**	−0.42**
HADS Dep	0.40 (0.59)	0.12	0.27	4.05 (0.99)	0.15	0.20	−14.02 (38)***	−0.35*	−0.30
CD-RISC	31.10 (1.11)	−0.01	−0.08	24.45 (1.50)	−0.28	−0.26	15.86 (38)***	0.36*	0.33*
SWLS	29.30 (2.55)	0.07	−0.03	23.00 (1.89)	−0.06	−0.13	8.85 (38)***	0.37*	0.31
SHS	27.20 (2.21)	0.31	0.33	21.10 (1.74)	0.14	0.18	9.67 (38)***	0.48**	0.48**
SCS Self-kindness	5.40 (0.68)	0.27	0.19	4.45 (0.82)	0.05	0.19	3.97 (38)***	0.36*	0.37*
SCS Humanity	5.35 (1.08)	0.40	0.37	3.90 (0.71)	0.09	0.13	4.97 (38)***	0.47**	0.46**
SCS Mindfulness	4.80 (1.10)	0.08	0.12	3.75 (0.63)	−0.18	−0.19	3.67 (38)***	0.22	0.23

*SD* standard deviation, *r* Pearson’s coefficient, *t(df)* t-contrast and degrees of freedom, *MTL* median telomere length, *20th* telomere length below which 20 % of the observed telomeres fall, *MAAS* Mindful Attention Awareness Scale, *FFMQ* Five Facet Mindfulness Questionnaire, *AAQ-2* Acceptance and Action Questionnaire, *HADS* Hospital Anxiety Depression Scale, *Anx* anxiety, *Dep* depression, *CD-RISC* Connor-Davidson Resilience Scale, *SWLS* Satisfaction with Life Scale, *SHS* Subjective Happiness Scale, *SCS* Self-Compassion Scale

*p<0.05; **p<0.01; ***p<0.001 (bilateral)

^a^Total sample

### Telomeres and Mindfulness Variables

Regarding the sociodemographic variables, only age showed significant relationships with MTL (*r* = 0.66; *p* < 0.001) and 20th percentile (*r* = 0.64; *p* < 0.001). The raw correlations between the two references of telomere measurements and the psychological variables supported the study’s hypothesis, as these variables were associated in the expected direction (Table 2). Of particular importance was the high correlation shown between telomere length and experiential avoidance (*r* = −0.53; *p* < 0.001). Notably, only three mindfulness subscales did not show a significant association with telomeres: nonjudging, nonreactivity, and self-compassion mindfulness. To determine which of the mindfulness variables was responsible for the telomere maintenance, we computed a regression analysis with regard to MTL (Table 3). We used a stepwise regression model (because of the limited statistical power) with the following variables: group (Zen/comparator), age (the only sociodemographic variable with a significant association with telomere length) and all of the psychological variables that showed a significant association with telomere length. As a result, only the variables that best explained the variability in telomere length were included in the final model, as the variables that did not add new information to the variables introduced in previous steps were removed.

**Table 3:** Regression models regarding the MTL and the 20th percentile (*N* = 40)

Variables	Adj-*R* ^2^	*F* (*df*_1_/*df*_2_) *p* ^a^	Se	DW	*p* ^b^
MTL	0.73	35.78 (1/36) <0.001	0.54	2.03	0.923
	*R*	B (95 % CI)	Se	Beta	*p* ^c^
Intercept		14.73 (12.77 to 16.69)	0.97		<0.001
Age	−0.80	−0.08 (−0.10 to −0.06)	0.01	−0.67	<0.001
AAQ-II	−0.46	−0.08 (−0.13 to −0.03)	0.03	−0.35	0.004
SCS Humanity	0.37	0.23 ( 0.03 to 0.44)	0.10	0.27	0.024
Variables	Adj-R^2^	*F* (*df*_1_/*df*_2_) *p* ^a^	Se	DW	*p* ^b^
20th percentile	0.67	26.85 (1/36) <0.001	0.29	2.02	0.596
	*R*	B (95 % CI)	Se	Beta	*p* ^c^
Intercept		6.94 ( 5.88 to 8.00)	0.52		<0.001
Age	−0.76	−0.04 (−0.05 to −0.03)	0.01	−0.65	<0.001
AAQ-II	−0.37	−0.03 (−0.06 to −0.01)	0.01	−0.29	0.024
SCS Humanity	0.36	0.12 (0.01 to 0.23)	0.05	0.29	0.029

Method: stepwise

Method: stepwise; *Adj-R* ^*2*^ adjusted coefficient of multiple determination, *Se* standard error, *DW* Dubin-Watson value, *R* partial correlation coefficient, *B* regression slope, *CI* confidence interval, *Beta* standardized slope, *AAQ-2* Acceptance and Action Questionnaire, *SCS*: Self-Compassion Scale

^a^ *p* Value for variance analysis associated with the regression

^b^ *p* Value for K-S test for normality contrast on residuals

^c^ *p* Value of Wald test result

Surprisingly, the components that measured mindfulness skills in the MAAS and FFMQ scales did not contribute to the final model. However, the following three factors made significant contributions: age (beta = −0.67; *p* < 0.001), experiential avoidance (beta = −0.35; *p* = 0.004), and the common humanity subscale from the self-compassion scale (beta = 0.27; *p* = 0.024). Next, a second regression model was conducted using the 20th percentile telomere values as the reference. The results were similar to those shown in the first model. Age (beta = −0.65; *p* < 0.001), experiential avoidance (beta = −0.29; *p* = 0.024) and the common humanity subscale of the self-compassion scale (beta = 0.29; *p* = 0.029) contributed significantly (Table 3).

## Discussion

The present data demonstrated that the expert meditators had a significantly longer MTL as well as lower percentages of short telomeres in their cells than the nonmeditator comparison group. This finding extends a small but growing body of literature showing longer telomeres (Schutte and Malouff 2014; Epel et al. 2009a, b; Conklin et al. 2015; Jacobs et al. 2011) and increases in telomerase (Lengacher et al. 2014) related to the practice of mindfulness. The ability to maintain longer telomeres through practicing meditation has many implications on health. The possible pathway between meditation and telomere length seems to be that (Schutte and Malouff 2014) mindfulness leads to individuals experiencing less stress, anxiety, and depression, which are all thought to be associated with cortisol level, and this association seems to be associated with telomerase activity.

Previous research has utilized the qPCR method for telomere measurement, which only provides the mean (not median) telomere length value per cell or per sample. However, a unique aspect of our study is that we applied the HT Q FISH method and were able to measure not only the MTL but also the abundance of short telomeres, which is an important parameter for assessing telomere functionality. Moreover, previous studies have shown similar results for the practice of Loving Kindness Meditation (Salzberg 1995), a type of meditation practice that focuses on developing a positive intention, unselfish kindness and warmth toward all people (Shaku et al. 2014). However, this study assessed a Zen Buddhism practice. Thus, it seems that mindfulness is a protective factor for telomere length regardless of the type of meditation practiced. These results might also be expected because Zen meditation has already been related not only to improvements in quality of life, better mental health (Shaku et al. 2014), and alpha and theta activity in many brain regions (generally related to relaxation) (Chiesa 2009) but also to decreases in oxidative stress (Mahagita 2010) and the resiliency of mitochondria (Bhasin et al. 2013), which may help prevent the process of ageing.

However, the exact drivers of mindfulness and their relationship to telomere length remain unclear. According to our results, it is likely that acceptance (measured as the absence of experiential avoidance by the AAQ-2), a process that is specifically promoted by mindfulness in general and Zen meditation in particular, plays a key role. However, there is still debate over which concept the AAQ-2 truly measures; some authors believe (Wolgast 2014) that it assesses distress and not avoidance/acceptance.

Acceptance has been found to predict a wide range of quality of life outcomes, such as depression, anxiety, and general mental health. It has been successfully applied to several specific topics, such as pain, smoking, diabetes management, tinnitus, weight, and coping with epilepsy and psychotic symptoms (Bond et al. 2011). However, this study is one of the first to show that acceptance is related to telomere length. Undoubtedly, from a cellular perspective, our results strengthen the idea that mental health and behavioural effectiveness are influenced more by how people relate to their thoughts and feelings than by the form of those thoughts or feelings (e.g., how negative they are).

Another factor that seems to play an essential role at the cellular level is one of the components of the self-compassion scale: common humanity. Persons with high anxiety have low mindfulness and compassion scores, and in these populations, mindfulness is a better predictor of disability than anxiety symptoms. This suggests that mindfulness can help protect against the feeling of being disabled by an anxiety disorder (Hoge et al. 2013a, b). Common humanity can also be viewed as a useful emotional regulation strategy in which painful or distressing feelings are not avoided but are held in awareness with kindness, understanding, and a sense of shared humanity.

Finally, it is necessary to emphasize that nearly all of the psychological variables (MAAS, AAQ, FFMQ, HADS, CD-RISC, SCS) were correlated with the telomere measures. No particular psychological variable had an association with telomere length that was clearly stronger than that of the others. Socioeconomic status did not show any relationship with telomere length despite being considered a relevant variable. In our sample, the meditators had a significantly lower salary than the comparison group because some of them were Buddhist monks who had taken vows of poverty; this fact may have biased the data.

This study has a number of limitations that suggest a cautious interpretation of the results. The most significant limitation of this research was its small sample size. Second, the processes examined are technically complex to measure, and in many ways, the instruments used are recent developments. Additional experience may lead us to refine these instruments, and different or improved instruments may reveal a different pattern of results. In addition, the participants were not randomly assigned to groups; thus, causality is unclear. Finally, telomere research is still in its early stages and potential variables that are difficult to measure and are currently unknown may alter the results.
